# Endovascular treatment of bilateral intracranial vertebral artery aneurysms: an algorithm based on a 10-year neurointerventional experience

**DOI:** 10.1136/svn-2020-000376

**Published:** 2020-08-13

**Authors:** Yisen Zhang, Zhongbin Tian, Wei Zhu, Jian Liu, Yang Wang, Kun Wang, Ying Zhang, Xinjian Yang, Wenqiang Li

**Affiliations:** 1 Department of Interventional Neuroradiology, Beijing Neurosurgical Institute and Beijing Tiantan Hospital, Capital Medical University, Beijing, China; 2 Department of Neurosurgery, First Affiliated Hospital of Nanchang University, Nanchang, Jiangxi, China

**Keywords:** aneurysm, dissection, flow diverter, intervention

## Abstract

**Background:**

The management of bilateral intracranial vertebral artery dissecting aneurysms (IVADAs) is controversial, and requires the development of endovascular treatment modalities and principles. We aim to investigate the endovascular treatment strategy and outcomes of bilateral IVADAs.

**Methods:**

We identified all bilateral IVADAs at a high-volume neurointerventional centre over a 10-year period (from January 2009 to December 2018). Radiographic and clinical data were recorded, and a treatment algorithm was derived.

**Results:**

Twenty-seven patients with bilateral IVADAs (54 IVADAs in total, 51 unruptured, 3 ruptured) were diagnosed. Four patients (14.8%) received single-stage endovascular treatment, 12 patients (44.4%) with staged endovascular treatment and 11 patients (40.8%) with unilateral endovascular treatment of bilateral IVADAs. Thirty-six IVADAs (85.7%) have complete obliteration at the follow-up angiography. Two of three ruptured IVADAs with stent-assisted coiling recanalised, and had further recoiling. Three patients (11.1%) have intraprocedural or postprocedural complications (two in single-stage and one in staged). Twenty-five patients (92.6%) had a favourable clinical outcome, and two patients (7.4%, all in single-stage) showed an unfavourable clinical outcome at follow-up. For the patients with unilateral reconstructive endovascular treatment, the contralateral untreated IVADAs were stable and had no growth or ruptured during follow-up period. None of all IVADAs had rebleeding during the clinical follow-up.

**Conclusions:**

Endovascular treatment can be performed in bilateral IVADAs with high technical success, high complete obliteration rates and acceptable morbidity/mortality. Contralateral IVADAs had low rates of aneurysm growth and haemorrhage when treated in a staged/delayed fashion.

## Introduction

The incidence of intracranial vertebral artery dissecting aneurysms (IVADAs) in the general population is very low at 0.001%–0.0015%,^1^ but associated risks of morbidity, mortality and rebleeding are high.^2–4^ Bilateral IVADAs account for less than 10% of all IVADAs, but their management is more complex and challenging.^5–9^ Endovascular treatment has emerged as a major therapeutic option for IVADAs, with successful results reported for staged bilateral vertebral artery (VA) occlusion of bilateral IVADAs;^10^ however, bilateral VA occlusion is not tolerated in many patients. Unilateral trapping or bilateral parent artery reconstructive therapy has been used to treat such intolerant patients. However, the occlusion of one VA may cause increased flow in the contralateral VA, with associated haemodynamic stress and possible contralateral aneurysmal rupture or recanalisation.^6 8 9^ Because of the limited number of bilateral IVADA cases reported to date, clinicians are accustomed to treating these pathologies based on personal or institutional experience, and consensus on how to treat bilateral IVADAs has not yet been reached. In this article, we detail our experience of bilateral IVADAs and propose an endovascular treatment protocol, which aims to achieve a more effective treatment of the condition.

## Methods

All medical data were reviewed retrospectively for diagnostic purposes.

### Patient selection and study population

Between January 2009 and December 2018, we reviewed clinical and radiologic data from our aneurysm database. A total of 8176 patients were referred to our department of interventional neuroradiology for endovascular treatment of a cerebral aneurysm. We identified 712 patients with 747 IVADAs; bilateral IVADAs had an incidence of 0.33% in all IVADAs patients at our centre. All patients included in this study met the following inclusion criteria: (1) bilateral IVADAs confirmed by digital subtraction angiography (DSA), CT angiography (CTA) or MR angiography (MRA); (2) the aneurysm in the patient was treated using an endovascular approach. The exclusion criteria included (1) a history of trauma or iatrogenic injury; (2) the presence of fibromuscular dysplasia; (3) the absence of any clinical follow-up. Ultimately, 27 patients with 54 IVADAs were identified. We collected information on patient demographics (age, sex and clinical history); rupture and configuration of IVADAs; endovascular treatment selected; treatment complications and angiographic and clinical follow-up outcomes.

### Endovascular treatment strategy

**Figure 1:**
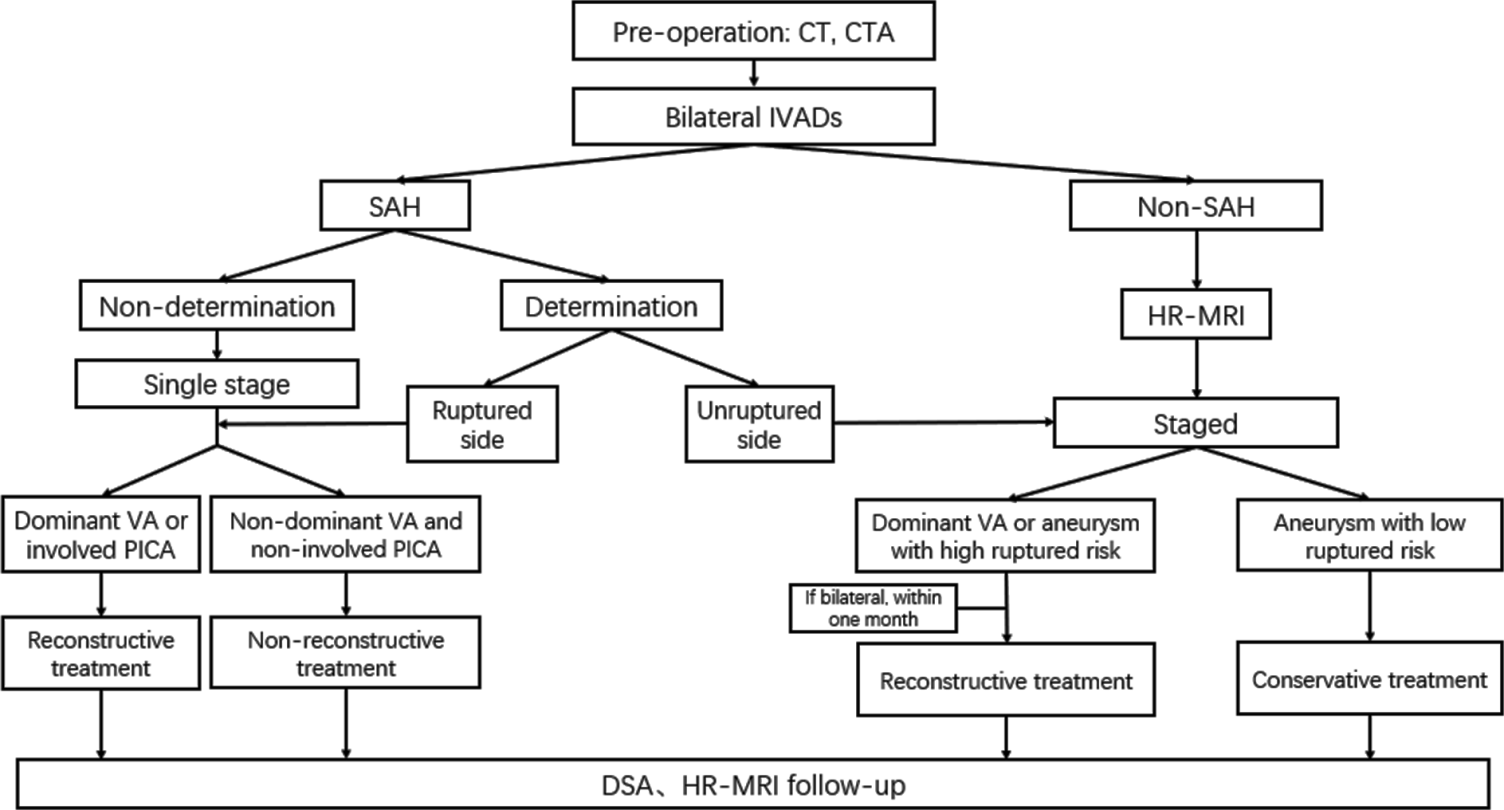
Algorithm showing endovascular treatment protocol for bilateral IVADAs. CTA, CT angiography; DSA, digital subtraction angiography; HR-MRI, high-resolution MRI; IVADAs, intracranial vertebral artery dissecting aneurysms; PICA, posterior inferior cerebellar artery; SAH, subarachnoid haemorrhage; VA, vertebral artery.

In our centre, two types of endovascular methods were used for treating IVADA patients, namely internal trapping and stent reconstructive techniques (stenting alone, stent-assisted coiling (SAC) techniques and flow diversion). The most important factors for selecting the treatment methods were the anatomic factors of the parent artery, including the involvement of the posterior inferior cerebellar artery (PICA) origin, the dominance of the affected VA and the sufficiency of the collateral blood supply. First, bilateral IVADA patients were classified into ruptured and unruptured, according to CT imaging data and patient symptoms. For patients with subarachnoid haemorrhage (SAH), one-stage treatment of bilateral IVADAs was performed for the patients whom the ruptured side was not confirmed. In contrast, a staged treatment was performed for the patients whom the ruptured side was confirmed. For patients with ruptured IVADAs, internal trapping was chosen as the treatment for the IVADA which was non-dominant or codominant, and which did not involve the PICA origin. The IVADA containing the dominant VA or involving the PICA origin was treated with stent reconstructive techniques. For the confirmed contralateral unruptured IVADAs with high ruptured risk, a staged treatment was performed 1 month later. For the unruptured patients, detailed image evaluation before treatment was performed, and staged endovascular treatment was chosen. Cases of IVADA with high rupture risk factors were treated first, such as aneurysms with dominant VA, complex aneurysms, aneurysm wall enhancement. If high rupture risk for bilateral IVADAs, the treatment of the contralateral IVADA was performed about 1 month later, otherwise, conservative treatment was considered. We optimised our management of bilateral IVADAs gradually after accumulating our experience, based on our previous work, we propose an endovascular treatment protocol for bilateral IVADAs during endovascular treatment (figure 1). For antiplatelet therapy before treatment, a loading dose of dual antiplatelet medication (300 mg aspirin and 300 mg clopidogrel) was given to patients with a ruptured IVADA who chose stent reconstructive techniques, while a standard antiplatelet regimen (100 mg aspirin and 75 mg clopidogrel; daily, from 5 days before the operation) was chosen for the unruptured patients.

All procedures were performed under general anaesthesia, and full procedural heparinization was used to achieve a targeted activated clotting time ranging from 250 to 300 s. Two catheters were used to deliver the coils for internal trapping, with the interlocking of the coils preventing coil migration during the procedure. For reconstructive treatment, we inserted the guiding catheter into the VA inferior to the atlas. For traditional stent alone technique, one or two self-expanding stents (Neuroform, Boston Scientific; Enterprise, Codman Neurovascular; Solitaire AB, ev3; or LVIS, MicroVention) were inserted, covering the entire segment of the dissecting aneurysm. For SAC, the stent delivery catheter was placed at the portion distal to the dissection, and a microcatheter was superselected to the IVADA. After filling two to three coils, the stent was released. For flow diversion, we adopted a triaxial supporting system to access the aneurysm, with the Pipeline Embolization Device (PED; Medtronic) introduced through a Marksman microcatheter. PED were delivered to reconstruct the parent artery in a satisfactory position, and then deployed. After the traditional stent procedure, 75 mg clopidogrel was administered every day for 6 weeks and 100 mg aspirin was administered each day for 6 months. After flow diversion, clopidogrel treatment was prolonged to 3 months, and aspirin prolonged to at least 1 year.

### Follow-up

Follow-up angiographic examination was performed with a conventional DSA or CTA scan at 3 months. The patient was followed up with an MRA scan annually. Patient outcome was measured using the modified Rankin Scale (mRS) score through neurologic examination at follow-up visits, and assess the neurological status of the patient during a telephone interview. The results were categorised into favourable outcomes (mRS, 0–2) and unfavourable outcomes (mRS, 3–6) based on the last clinical follow-up at the time of the study.

## Results

The clinical, radiographic characteristics, treatment methods and outcomes of the bilateral IVADA cases are summarised in tables 1–3. The mean age of the 27 patients (4 women and 23 men) was 54.26±6.22 years (mean±SD). Six patients (22.2%) presented with incidental findings; 16 patients (59.3%) presented with different symptoms (11 with headache, 4 with dizziness, 1 with numbness of limbs). All IVADAs were successfully treated with endovascular treatment. Thirty-six IVADAs (85.7%) have complete obliteration at the follow-up angiography, and two IVADAs (4.8%) recanalised which had further retreatment. Three patients (11.1%) have intraprocedural or postprocedural complications. None had bleeding or rebleeding during the clinical follow-up. Twenty-five patients (92.6%) had a favourable clinical outcome at the follow-up, and two patients (7.4%) showed an unfavourable clinical outcome at the last follow-up. According to the treatment method used, the included patients were divided into three categories, namely (1) single-stage, (2) staged and (3) unilateral endovascular treatment.

**Table 1:** Clinical and radiographic characteristics of bilateral IVADAs with single-stage endovascular treatment

Case no.	Presentation	Side, PICA-involving, ruptured	Configuration	H-H	Admission mRS score	Treatment	Immediate angiography*	Complication (days)	Follow-up angiography* (months)	Last mRS score (months)
1	Acute headache	R, involving, not determined L, involving, not determined	Pearl and string sign Fusiform	Ⅱ	4	SAC SAC	Near-complete obliteration Near-complete obliteration	–	Recanalisation (3)→recoiling→ stable (6) Complete obliteration (3)	1 (6)
2	Headache	L, no involving, unruptured R, no involving, unruptured	Pearl and string sign Fusiform	–	1	SAC Trapping	Near-complete obliteration Complete obliteration	Muscle weakness of left limbs (3 days)	Complete obliteration (6) Complete obliteration (6)	3 (48)
3	Dizziness	R, no involving, unruptured L, no involving, unruptured	Pearl and string sign Pearl and string sign	–	1	SAC SAC	Complete obliteration Complete obliteration	Sudden unconsciousness (2 months)	Complete obliteration (2) Complete obliteration (2)	3 (36)
4	Incidence	R, no involving, unruptured L, no involving, unruptured	Pearl and string sign Fusiform	–	0	SAC Stent alone	Complete obliteration Partial obliteration	–	Complete obliteration (6) Complete obliteration (6)	1 (30)

*Angiography results are classified as follows: stable, no interval changes of the dissecting aneurysm in size or shape; complete obliteration, complete occlusion of the dissecting aneurysm sac with reconstruction of the parent artery; near-complete obliteration, neck remnant of the dissecting aneurysm.

H-H, Hunt-Hess; IVADAs, intracranial vertebral artery dissecting aneurysms; L, left; mRS, modified Rankin Scale; PICA, posterior inferior cerebellar artery; R, right; SAC, stent-assisted coiling.

**Table 2:** Clinical and radiographic characteristics of bilateral IVADAs with staged endovascular treatment

Case no.	Presentation	Side, PICA-involving, ruptured	Configuration	H-H	Admission mRS score	First treatment				Interval time (months)	Second treatment				Last mRS score (months)
						Side, treatment	Immediate angiography*	Follow-up angiography* (months)	Complication		Side, treatment	Immediate angiography*	Follow-up angiography*	Complication (months)	
1	Acute headache	R, involving, ruptured L, no involving, unruptured	Pearl and string sign Pearl and string sign	Ⅱ	4	R, SAC	Near-complete obliteration	Recanalisation (1)→recoiling→stable (7)	–	1	L, SAC	Near-complete obliteration	Complete obliteration (6)	–	1 (7)
2	Headache	L, no involving, unruptured R, no involving, unruptured	Fusiform Pearl and string sign	–	1	R, SAC	Near-complete obliteration	Complete obliteration (3)	–	3	L, SAC	Complete obliteration	Complete obliteration (6)	–	0 (9)
3	Numbness of limbs	L, no involving, unruptured R, no involving, unruptured	Fusiform Fusiform	–	2	L, stent alone	Partial obliteration	Complete obliteration (9)	–	3	R, stent alone	Partial obliteration	Complete obliteration (6)	–	1 (9)
4	Incidence	L, no involving, unruptured R, no involving, unruptured	Pearl and string sign Pearl and string sign	–	0	R, SAC	Complete obliteration	Complete obliteration (9)	–	3	L, SAC	Complete obliteration	Complete obliteration (6)	–	0 (9)
5	Headache	L, no involving, unruptured R, no involving, unruptured	Pearl and string sign Fusiform	–	1	L, SAC	Complete obliteration	Complete obliteration (10)	–	3	R, SAC	Near-complete obliteration	Complete obliteration (7)	–	0 (9)
6	Headache	L, involving, unruptured R, involving, unruptured	Fusiform Fusiform	–	1	L, stent alone	Partial obliteration	Near-complete obliteration (1)	–	1	R, stent alone	Partial obliteration	Stable (3)	–	0 (9)
7	Dizziness	L, involving, unruptured R, involving, unruptured	Fusiform Fusiform	–	2	R, stent alone	Partial obliteration	Complete obliteration (6)	–	3	L, stent alone	Partial obliteration	Stable (3)	–	0 (6)
8	Headache	R, involving, unruptured L, involving, unruptured	Fusiform Fusiform	–	1	L, PED alone	Partial obliteration	Complete obliteration (6)	–	2.5	R, PED alone	Partial obliteration	Complete obliteration (3.5)	–	0 (6)
9	Dizziness	R, involving, unruptured L, involving, unruptured	Pearl and string sign Fusiform	–	1	L, PED alone	Partial obliteration	Complete obliteration (9)	–	2	R, PED alone	Partial obliteration	Complete obliteration (7)	–	0 (9)
10	Dizziness	R, involving, unruptured L, involving, unruptured	Pearl and string sign Fusiform	–	1	R, PED alone	Partial obliteration	Complete obliteration (9)	–	3	L, PED alone	Partial obliteration	Complete obliteration (6)	Parent artery occlusion (6)	0 (9)
11	Headache	R, no involving, unruptured L, no involving, unruptured	Pearl and string sign Fusiform	–	1	R, SAC	Complete obliteration	Complete obliteration (7)	–	2	L, PED alone	Partial obliteration	Complete obliteration (6)	–	0 (8)
12	Headache	R, no involving, unruptured L, involving, unruptured	Fusiform Fusiform	–	1	R, PED alone	Partial obliteration	Complete obliteration (7)	–	1	L, PED alone	Partial obliteration	Complete obliteration (6)	–	0 (7)

*Angiography results are classified as follows: stable, no interval changes of the dissecting aneurysm in size or shape; complete obliteration, complete occlusion of the dissecting aneurysm sac with reconstruction of the parent artery; near-complete obliteration, neck remnant of the dissecting aneurysm; partial resolution, any opacification of the dissecting aneurysm sac.

H-H, Hunt-Hess; IVADAs, intracranial vertebral artery dissecting aneurysms; L, left; mRS, modified Rankin Scale; PED, Pipeline Embolization Device; PICA, posterior inferior cerebellar artery; R, right; SAC, stent-assisted coiling.

**Table 3:** Clinical and radiographic characteristics of bilateral IVADAs with unilateral endovascular treatment

Case no.	Presentation	Side, PICA-involving, ruptured	Configuration	H-H	Admission mRS score	Treatment	Immediate angiography*	Complication	Follow-up angiography* (months)	Last mRS score (months)
1	Dizziness	L, no involving, unruptured R, no involving, unruptured	Pearl and string sign Fusiform	–	1	SAC –	Near-complete obliteration –	–	Complete obliteration (6) Stable (6)	0 (6)
2	Headache	L, no involving, unruptured R, no involving, unruptured	Pearl and string sign Fusiform	–	2	SAC –	Near-complete obliteration –	–	Complete obliteration (8) Stable (6)	0 (8)
3	Headache	L, no involving, unruptured R, no involving, ruptured	Fusiform Pearl and string sign	Ⅰ	3	– SAC	– Near-complete obliteration	–	Stable (3) Stable (3)	0 (6)
4	Dizziness	L, no involving, unruptured R, involving, unruptured	Pearl and string sign Pearl and string sign	–	1	– SAC	– Near-complete obliteration	–	NA	0 (6)
5	Dizziness	L, no involving, unruptured R, no involving, unruptured	Fusiform Pearl and string sign	–	1	– SAC	– Near-complete obliteration	–	Stable (6) Complete obliteration (6)	0 (6)
6	Dizziness	L, no involving, unruptured R, no involving, unruptured	Pearl and string sign Fusiform	–	1	SAC –	Complete obliteration –	–	Complete obliteration (6) Stable (6)	0 (3)
7	Incidence	L, no involving, unruptured R, no involving, unruptured	Fusiform Fusiform	–	0	SAC –	Complete obliteration –	–	Complete obliteration (6) Stable (6)	0 (6)
8	Incidence	L, involving, unruptured R, no involving, unruptured	Fusiform Pearl and string sign	–	0	PED alone –	Partial obliteration –	–	Complete obliteration (3) Stable (3)	0 (3)
9	Incidence	L, involving, unruptured R, no involving, unruptured	Fusiform Fusiform	–	0	PED alone –	Partial obliteration –	–	Complete obliteration (6) Stable (6)	0 (6)
10	Incidence	L, no involving, unruptured R, involving, unruptured	Fusiform Fusiform	–	0	– PED alone	– Partial obliteration	–	Stable (6) Complete obliteration (6)	0 (6)
11	Dizziness	L, no involving, unruptured R, no involving, unruptured	Fusiform Fusiform	–	0	PED alone –	Partial obliteration –	–	Complete obliteration (5) Stable (5)	0 (5)

*Angiography results are classified as follows: stable, no interval changes of the dissecting aneurysm in size or shape; complete obliteration, complete occlusion of the dissecting aneurysm sac with reconstruction of the parent artery; near-complete obliteration, neck remnant of the dissecting aneurysm, partial resolution, any opacification of the dissecting aneurysm sac.

H-H, Hunt-Hess; IVADAs, intracranial vertebral artery dissecting aneurysms; L, left; mRS, modified Rankin Scale; NA, not applicable; PED, Pipeline Embolization Device; PICA, posterior inferior cerebellar artery; R, right; SAC, stent-assisted coiling.

### Single-stage endovascular treatment

**Figure 2:**
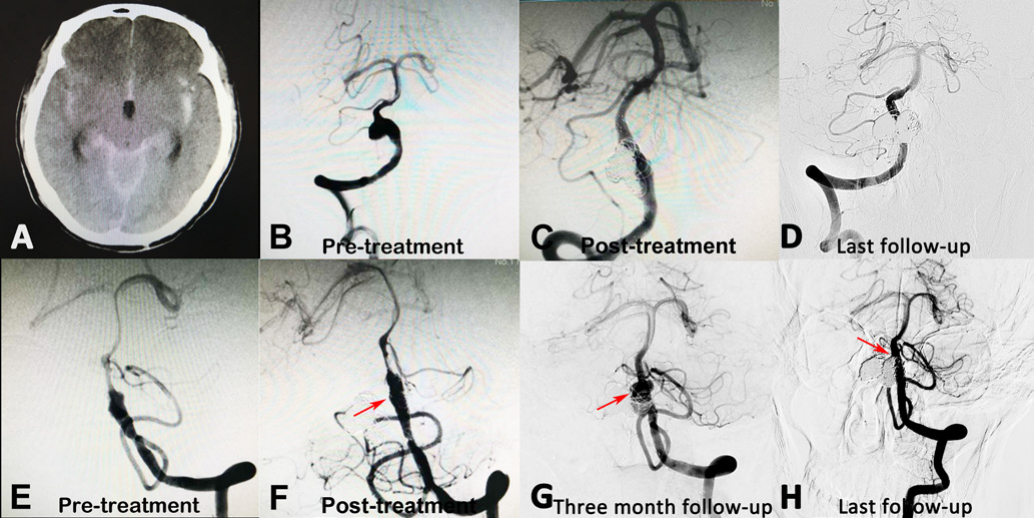
(A) A patient showing subarachnoid haemorrhage on CT imaging. (B and E) Digital subtraction angiography showed bilateral IVADAs. The ruptured side could not be determined based on the CT scan. (C and F) Thus, for this ruptured patient, both aneurysms were treated with stent-assisted coil embolisation at one stage, and a near-complete obliteration was achieved. (G) At the 3-month follow-up, the left IVADA recanalised, and the right IVADA was stable. The recanalised IVADA was further retreated with recoiling. (D and H) Both IVADAs were stable at the last follow-up angiography. IVADAs, intracranial vertebral artery dissecting aneurysms.

Four patients received single-stage endovascular treatment (table 1). Seven IVADAs (87.5%) have complete obliteration at the follow-up angiography, and one IVADA (12.5%) in a patient with SAH recanalised at the 3-month follow-up. Both IVADAs of the patient with SAH were treated with SAC, for which a CT scan was unable to determine the ruptured side. At first follow-up, the right IVADA was stable, the recanalised left IVADA was treated again with coiling. Both IVADAs were complete obliteration at the final follow-up angiography (figure 2). Two patients (50%) had complications, and had unfavourable results at last follow-up. One patient was treated with trapping of the right IVADA and SAC of the left side. The patient displayed muscle weakness in their right limbs 3 days after treatment, and the postprocedure MRI showed cerebellar infarction. One patient with SAC of both IVADAs did not fully comply with the medical advices at 2 months postprocedure, and stop taking prescribed antiplatelet medications. The patient showed sudden unconsciousness (mRS=5). The MRI showed cerebellar infarction and stenosis of the vertebrobasilar artery. The patient received tissue-type plasminogen activator thrombolysis intravenously, and recovered to consciousness (mRS=4). The mRS score of both patients was 3 at last follow-up.

### Staged endovascular treatment

**Figure 3:**
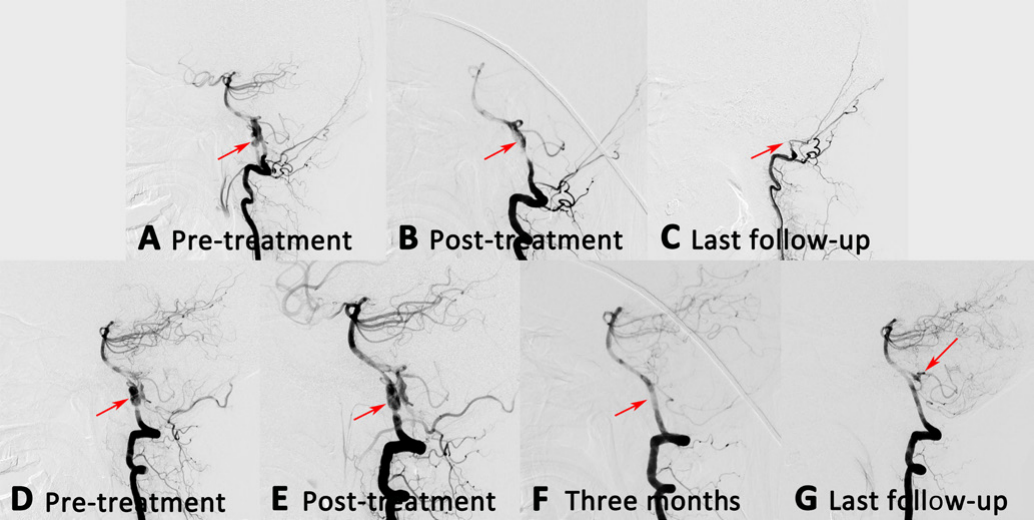
(A and D) An unruptured patient with bilateral IVADAs. This patient received a two-stage treatment. (E) At the first stage, the right aneurysm was treated with PED alone, and partial obliteration was achieved immediately. (F) Three months later, the right aneurysm was completely obliterated, while no compensatory blood flow was supplied to the left vertebral artery. (B) At the same time, the left aneurysm was also treated with PED alone, and partial obliteration was achieved immediately. (G) At the last follow-up DSA, the right aneurysm remained stable. The left posterior inferior cerebellar artery received compensatory blood flow from the right vertebral artery, while the left parent artery disappeared (C). No clinical symptoms were found. DSA, digital subtraction angiography; IVADAs, intracranial vertebral artery dissecting aneurysms; PED, Pipeline Embolization Device.

Twelve patients received staged endovascular treatment, and showed in table 2. Twenty IVADAs (83.3%) have complete obliteration at the follow-up angiography. One ruptured IVADA (4.2%) recanalised, and had retreatment. It remained stable at further follow-up. The unruptured IVADA of the patient was treated staged within 1 month, and showed complete obliteration at the final follow-up. For the 11 unruptured patients, 13 IVADAs (59.0%) were reconstructed with traditional stent, and nine IVADAs were reconstructed with flow diverter (41.0%). Complete obliteration rate was achieved 100% in IVADAs with flow diverter and 76.9% in IVADAs with traditional stent at follow-up angiography. Eleven IVADAs (50.0%) involved the origin of PICA, six IVADAs were with flow diverter. One complication occurred in the patient of IVADA involved PICA with flow diverter, the parent artery showed stenosis after flow diverter, and occluded at follow-up period. However, no clinical symptoms occurred, for which the PICA received compensatory blood flow from the other VA (figure 3). All patients (100%) had favourable clinical outcome at the follow-up.

### Only one side of bilateral IVADAs with endovascular treatment

**Figure 4:**
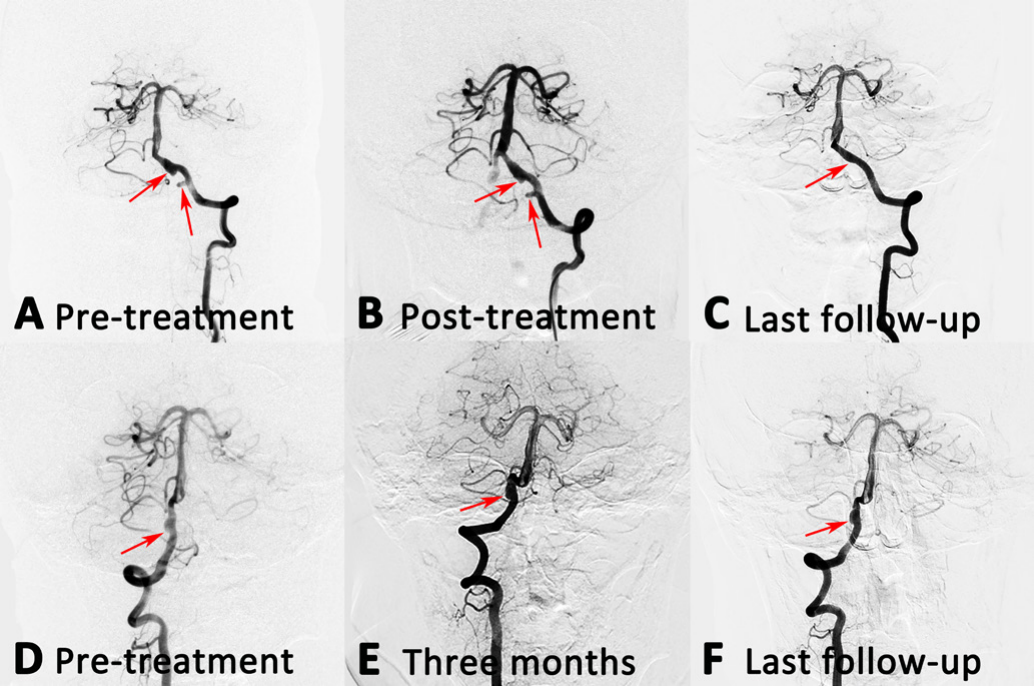
(A and D) A patient with bilateral IVADAs, who was treated only one side. (B) The left aneurysm was treated with PED alone, and partial obliteration was achieved immediately. (E) Three months later, the right aneurysm remained stable. (C and F) At the last follow-up angiography, the left aneurysm was completely obliterated, and the right aneurysm remained stable. IVADAs, intracranial vertebral artery dissecting aneurysms; PED, Pipeline Embolization Device.

Eleven patients received only unilateral endovascular treatment, and showed in table 3. One patient had a ruptured IVADA and a Hunt-Hess grade of 1, and three had IVADAs involving the origin of PICA. Four IVADAs (36.4%) were reconstructed with flow diverter. At follow-up angiography, complete obliteration was achieved in all IVADAs (100%) with flow diverter, and the complete obliteration rate was 71.4% in six IVADAs with SAC for that one patient refused to return for the invasive inspection. No procedure-related complications occurred, and all patients had favourable clinical outcome during the follow-up period. One tandem IVADA had complete obliteration after flow diverter, and the contralateral untreated IVADA were stable on follow-up angiography (figure 4).

### Comparison of three different treatments in patients with unruptured bilateral IVADAs

We compared the treatments in patients with bilateral IVADAs, obviously significant difference was showed in treatment methods (table 4). Importantly, the patients with staged treatment and one side reconstructive treatment had significant lower thromboembolic complication rate compared with the patients with single-stage treatment (p<0.01 for both comparisons). At admission, the mRS score had no difference between three methods. However, for the last mRS score, the patients with staged treatment and one side reconstructive treatment were significantly lower than that with single-stage treatment (p<0.01 for both comparisons).

**Table 4:** Comparison of the unruptured IVADAs with three different treatments

Variables	Single-stage treatment (A), n=6	Staged treatment (B), n=22	P value (A vs B)	One side treatment (C), n=20	P value (A vs C)	P value (B vs C)
Admission mRS score	0.67±0.58	1.09±0.54	0.24	0.60±0.70	0.78	0.07
Treatment method			0.05		0.01*	<0.01*
Conservative	0 (0)	0 (0)		10 (50.0)		
Trapping	1 (16.7)	0 (0)		0 (0)		
SAC	4 (66.7)	7 (31.8)		6 (30.0)		
Stent alone	1 (16.7)	6 (27.3)		0 (0)		
Flow diverter	0 (0)	9 (40.9)		4 (20.0)		
Thromboembolic complications	2 of 3 (66.7)	0 of 11 (0)	<0.01*	0 of 10 (0)	<0.01*	–
Immediate angiography			0.07		0.18	0.11
Partial obliteration	1 (16.7)	15 (68.2)		4 (40.0)		
Near-complete obliteration	1 (16.7)	2 (9.1)		4 (40.0)		
Complete obliteration	4 (66.7)	5 (22.7)		2 (20.0)		
Follow-up angiography			0.12		0.81	0.06
Improve	2 (33.3)	15 (68.2)		7 (38.9)		
Stable	4 (66.7)	7 (31.8)		11 (61.1) *		
Recanalisation	0 (0)	0 (0)		0 (0)		
Last mRS score	2.33±1.16	0.09±0.30	<0.01*	0±0	<0.01*	0.34

*One patient with bilateral IVADs had no follow-up.

IVADAs, intracranial vertebral artery dissecting aneurysms; mRS, modified Rankin Scale; SAC, stent-assisted coiling.

## Discussion

Of the patients who received the single-stage endovascular treatment, one had a cerebellar infarction, and insufficient compensatory blood flow might be the reason. Moreover, urgent in-stent thrombosis was observed in an uncomplied patient. Both of the patients have unfavourable outcomes at last follow-up. Similarly, one patient with staged treatment had parent artery occlusion at follow-up, but no clinical symptoms occurred. In the patients receiving staged or unilateral endovascular treatment, all IVADAs were reconstructed using stent alone, using SAC or flow diverter, and no growth and ruptured IVADAs occurred at follow-up. These results may indicate that the haemodynamic alteration of the contralateral VA was slight after unilateral reconstructive endovascular treatment, and as such, did not lead to contralateral IVADA enlargement and possible rupture. Compared with the three methods, the patients with staged treatment and one side reconstructive treatment had significant lower thromboembolic complication rate and mRS score compared with the patients with single-stage treatment. Such results might demonstrate that the application of staged reconstructive endovascular treatment in patients with unruptured bilateral IVADAs might decrease the risk of thromboembolic events and led to favourable outcomes. For the ruptured IVADAs, the best treatment might be parent artery occlusion, for which a lower recanalisation rate was shown. However, trapping the parent artery increased haemodynamic stress on the contralateral VA, leading to enlargement and possibly to rupture. In this study, a reconstructive treatment was performed on three ruptured IVADAs, of which two were recanalised. A close follow-up and treatment again with coiling might be important for such aneurysms.

### Endovascular treatment of bilateral IVADAs presenting with SAH

IVADA presenting with SAH has been reported to occur in approximately 3% of all cases of SAH. As this condition has been known to cause a high rate of rebleeding, prompt non-reconstructive treatment procedures, such as proximal occlusion or trapping, are advocated to prevent this.^6 11^ Open surgical intervention might be not a good option for acute dissecting aneurysms of the VA, given the rather high risks of treatment-related morbidity and mortality; endovascular non-reconstructive treatment is the standard procedure.^12^ However, if the contralateral VA shows hypoplasia or occlusion, or if the IVADA itself is of the PICA-involved type, proximal occlusion or trapping are inadequate. In this case, open surgical might also be considered even though it is invasive and technically difficult. Saito *et al* 13 and Ota *et al* 14 reported that bypass of arteries for the bilateral IVADAs was an effective method to preserve the perforators and provide blood flow. Furthermore, trapping the VA places increased haemodynamic stress on the contralateral VA, which might lead to enlargement and possibly rupture.^15 16^ Therefore, revascularisation of the parent (ipsilateral) artery territory can be performed to preserve ipsilateral VA flow.^17–19^ In our study, three patients with SAH were treated with parent artery reconstruction, while two IVADAs recanalised at follow-up. Such recanalised IVADAs were also reported to have a high rate of rebleeding associated with high risk of morbidity and mortality. For the recanalised IVADAs in this study, patients were treated again with coiling and complete obliteration was found at follow-up.

Detection of the ruptured side is not easy in cases of bilateral IVADAs presenting with SAH.^18^ The following determinants have been identified in prior reports: the headache side;^20^ hemiplegia;^21^ a thicker haematoma on a CT image;^22 23^ a larger and more irregular aneurysm;^21 22^ the pearl-and-string sign;^21^ a pooling of contrast media in a pseudoaneurysm on angiography^22 23^ and an intramural haematoma on MR images.^24^ If the ruptured side is not clearly verified, bilateral, single-stage treatment should be performed to avoid the misidentified lesion being untreated. In our study, the laterality of the SAH on CT scans and the pooling of the contrast agent on the cerebral angiography clearly showed the ruptured side in two cases. However, in one case, the ruptured side could not be determined, and we reconstructed the bilateral IVADAs in a single stage; fortunately, no procedure-related complication occurred in this patient. Thus, in our patients, we first determined the ruptured side of the IVADA based on the laterality of the SAH on CT scans. Reconstructive endovascular treatment was our first choice for a ruptured IVADA with a dominant VA or with involvement of the PICA, while non-reconstructive endovascular treatment, such as proximal occlusion or trapping, was performed for a ruptured IVADA without these characteristics. For confirmed cases of a ruptured IVADA, endovascular treatment of bilateral IVADAs should be performed, and staged reconstructive endovascular treatment should be performed for an unruptured IVADA.

### Single-stage or staged endovascular treatment for bilateral IVADAs?

With recent advances in device technologies, endovascular treatment has become the first-line treatment for VA dissecting aneurysms. Reconstructive endovascular treatment preserves bilateral VA flow, while treatment by stent placement can ensure preservation of the VA. Stent-assisted coil embolisation is better than stent-alone therapy for promoting faster thrombosis, and it is simpler and more acceptable for treating both aneurysms in a single session than trapping, because it does not require a balloon occlusion test and can be performed on patients with no tolerance. In addition, single-stage endovascular treatment has potential advantages, in which dual antiplatelet therapy is mandatory. Securing all aneurysms theoretically decreases the fatal bleeding risk in case of rupture of the unsecured aneurysms. The duration of antiplatelet therapy is reduced in single-stage treatment compared with staged procedures. However, the use of stents or flow diverters increases the risk of thromboembolic events. In particular, in patients with bilateral IVADAs, single-stage treatment with flow diverters carries the potential risk of simultaneous stent thrombosis, which may cause morbidity and mortality by blocking the collateral pathways. Wilkinson *et al* 25 reported staged endovascular treatment of bilateral IVADAs with SAH, in which the IVADAs were treated successfully by staged SAC. First, a ruptured IVADA was treated, followed by subsequent treatment of the contralateral unruptured IVADA; no thromboembolic events occurred. Moreover, Andic *et al* 26 also found that staged treatment might be used instead of single-stage treatment to avoid thromboembolic events in the patients with bilateral intracranial aneurysms. In the current study, two patients with bilateral IVADAs were treated simultaneously in a single stage, and have unfavourable clinical outcomes. One patient showed cerebellar infarction after treatment, and another patient have urgent in-stent thrombosis. Conversely, delayed mechanical stent occlusion caused the loss of the parent artery in a patient with staged treatment. No clinical symptoms were found during the follow-up period. Staging of endovascular treatment in this group of patients may be a reasonable choice in order to avoid thromboembolic events, despite leaving an aneurysm unsecured under dual antiplatelet therapy.

### Era of reconstructive endovascular treatment for bilateral IVADAs

To reduce unpredictable haemodynamic stress on the contralateral aneurysm, revascularisation of the parent (ipsilateral) artery territory is desirable. Endovascular techniques like flow-diverting stents have been used successfully to preserve the VA. In a recent study, Park *et al* 17 reported that stent-only therapy using single, double or triple overlapping stents was safe and effective for the treatment of IVADAs. In addition, multiple-overlapping, but not single, stent therapy, resulted in angiographic improvement.^19^ Stent application over the dissecting segment, especially with multiple overlapping stents, can lead to haemodynamic changes (decreases in inflow complexity, momentum, velocity and wall shear stress), whereas intra-aneurysmal blood turnover time is increased.^27 28^

Flow diverters have emerged as an attractive treatment option for these challenging lesions. These devices are placed in the parent artery and affect the haemodynamics such that there is an eventual remodelling of the affected segment via endothelial proliferation. Originally, flow diverters were used to treat wide-necked aneurysms of the anterior circulation.^29^ However, as interventionalists have become more experienced and comfortable with flow diverters, these devices have shown benefits for posterior circulation dissecting aneurysms and have yielded favourable outcomes.^30 31^ However, the use of flow diverters on the VA system is not without risks. In-stent thrombosis and thromboembolic events are feared complications, with up to 52% of patients showing changes on MR diffusion-weighted imaging scans and up to 14% experiencing symptomatic infarctions.^32^ Occlusion of perforators can occur acutely by direct mechanical blockage from a tine or strut of the flow diverter, by a thrombus from the device migrating to the branching artery, or chronically by excessive neointimal proliferation (ie, in-stent stenosis). The use of antiplatelet medications, close monitoring of platelet function with medication and dose adjustments based on response are important considerations for flow diverter placement.

As in our patient, reconstructive endovascular treatment can be used to exclude the IVADAs without obstructing their flow. This thereby avoids a dramatic increase in flow and haemodynamic stress in the contralateral IVADAs, which would otherwise likely contribute to high mortality. Ishikawa *et al* 33 reported a bilateral IVADAs case with SAH that the ruptured side was well treated with SAC, and the contralateral side was stable at follow-up after treatment. Such results might also support that reconstructive endovascular treatment has little impact on the haemodynamic stress to the contralateral IVADA. The staged reconstructive endovascular treatment of bilateral IVADAs appears to be an efficacious approach. Although in our patient the second IVADA was repaired one month after the first, the optimal timing for staged bilateral repair remains uncertain. Even stent-based therapy, however, causes some alteration in flow that may predispose the enlargement of a previously existing contralateral pseudoaneurysm, or even the formation of a new contralateral pseudoaneurysm.^34^ For this reason, follow-up shortly after the initial repair is important in order to be able to promptly recognise any alteration in the contralateral VA and to intervene prior to any catastrophic event.

### Limitations

Our study has several limitations, the study was a retrospective study over a long time-period, so that might include some confounding factors, such as patient selection and treatment technique bias. Bilateral IVADAs are rare entities, and this study enrolled only 25 such cases. Finally, given the rarity of bilateral IVADAs, the timespan over which the endovascular treatment occurred was long, and the methods used for the treatment were diverse are also limitations.

## Conclusion

The endovascular treatment protocol for bilateral IVADAs proposed in this study might be helpful in the management of this rare lesion. The endovascular treatment was safe and effective, and led to favourable outcomes for bilateral IVADAs. Application of staged reconstructive endovascular treatment might decrease the risk of thromboembolic events, and of contralateral IVADA growth and rupture. Flow diverters might enable a higher rate of complete occlusion embolisation for this rare lesion, while occlusion of the parent artery should also be considered.

## References

[1] Schievink WI Spontaneous dissection of the carotid and vertebral arteries. N Engl J Med 2001;344:898–906. 10.1056/NEJM200103223441206 11259724

[2] Yamada M, Kitahara T, Kurata A, et al Intracranial vertebral artery dissection with subarachnoid hemorrhage: clinical characteristics and outcomes in conservatively treated patients. J Neurosurg 2004;101:25–30. 10.3171/jns.2004.101.1.0025 15255247

[3] Mizutani T, Aruga T, Kirino T, et al Recurrent subarachnoid hemorrhage from untreated ruptured vertebrobasilar dissecting aneurysms. Neurosurgery 1995;36:905–13. 10.1227/00006123-199505000-00003 7791980

[4] Kim BM, Kim SH, Kim DI, et al Outcomes and prognostic factors of intracranial unruptured vertebrobasilar artery dissection. Neurology 2011;76:1735–41. 10.1212/WNL.0b013e31821a7d94 21576691

[5] Shin YS, Kim BM, Kim S-H, et al Endovascular treatment of bilateral intracranial vertebral artery dissecting aneurysms presenting with subarachnoid hemorrhage. Neurosurgery 2012;70:75–81. 10.1227/NEU.0b013e31822ed1f0 21796008

[6] Koh JS, Ryu CW, Lee SH, et al Bilateral vertebral-artery-dissecting aneurysm causing subarachnoid hemorrhage cured by staged endovascular reconstruction after occlusion. Cerebrovasc Dis 2009;27:202–4. 10.1159/000193464 19153480

[7] Yoon S-M, Shim J-J, Kim S-H, et al Bilateral vertebral artery dissecting aneurysms presenting with subarachnoid hemorrhage treated by staged coil trapping and covered stents graft. J Korean Neurosurg Soc 2012;51:155–9. 10.3340/jkns.2012.51.3.155 22639713 PMC3358603

[8] Inoue A, Kohno K, Takechi A, et al Bilateral vertebral artery dissecting aneurysm with subarachnoid hemorrhage treated with staged bilateral vertebral artery coil occlusion: a case report. Surg Neurol 2008;70:319–22. 10.1016/j.surneu.2007.04.019 18207505

[9] Lee DH, Yoon WK, Baik MW, et al The difference of each angiographic finding after multiple stent according to stent type in bilateral vertebral artery dissection. J Cerebrovasc Endovasc Neurosurg 2013;15:229–34. 10.7461/jcen.2013.15.3.229 24167805 PMC3804663

[10] Sakamoto S, Ohba S, Shibukawa M, et al Staged bilateral vertebral artery occlusion for ruptured dissecting aneurysms of the basilar artery: a report of 2 cases. Surg Neurol 2005;64:456–61. 10.1016/j.surneu.2005.01.021 16253701

[11] Kurata A, Ohmomo T, Miyasaka Y, et al Coil embolization for the treatment of ruptured dissecting vertebral aneurysms. AJNR Am J Neuroradiol 2001;22:11–18. 11158881 PMC7975530

[12] Rinne J, Hernesniemi J, Puranen M, et al Multiple intracranial aneurysms in a defined population: prospective angiographic and clinical study. Neurosurgery 1994;35:803–8. 10.1227/00006123-199411000-00001 7838326

[13] Saito N, Kamiyama H, Takizawa K, et al Management strategy for bilateral complex vertebral artery aneurysms. Neurosurg Rev 2016;39:289–96. 10.1007/s10143-015-0686-3 26564148

[14] Ota N, Tanikawa R, Eda H, et al Radical treatment for bilateral vertebral artery dissecting aneurysms by reconstruction of the vertebral artery. J Neurosurg 2016;125:953–63. 10.3171/2015.8.JNS15362 26848908

[15] Inui Y, Oiwa Y, Terada T, et al De novo vertebral artery dissecting aneurysm after contralateral vertebral artery occlusion--two case reports. Neurol Med Chir 2006;46:32–6. 10.2176/nmc.46.32 16434824

[16] Zhao K-J, Fang Y-B, Huang Q-H, et al Reconstructive treatment of ruptured intracranial spontaneous vertebral artery dissection aneurysms: long-term results and predictors of unfavorable outcomes. PLoS One 2013;8:e67169. 10.1371/journal.pone.0067169 23840616 PMC3693966

[17] Park SI, Kim BM, Kim DI, et al Clinical and angiographic follow-up of stent-only therapy for acute intracranial vertebrobasilar dissecting aneurysms. AJNR Am J Neuroradiol 2009;30:1351–6. 10.3174/ajnr.A1561 19342544 PMC7051581

[18] Kono K, Shintani A, Fujimoto T, et al Stent-assisted coil embolization and computational fluid dynamics simulations of bilateral vertebral artery dissecting aneurysms presenting with subarachnoid hemorrhage: case report. Neurosurgery 2012;71:E1192–201. 10.1227/NEU.0b013e318270603a 22948198

[19] Kim BM, Shin YS, Kim S-H, et al Incidence and risk factors of recurrence after endovascular treatment of intracranial vertebrobasilar dissecting aneurysms. Stroke 2011;42:2425–30. 10.1161/STROKEAHA.111.617381 21778439

[20] Caplan LR, Baquis GD, Pessin MS, et al Dissection of the intracranial vertebral artery. Neurology 1988;38:868–77. 10.1212/WNL.38.6.868 3368067

[21] Otawara Y, Ogasawara K, Ogawa A, et al Dissecting aneurysms of the bilateral vertebral arteries with subarachnoid hemorrhage: report of three cases. Neurosurgery 2002;50:1372–4. 10.1097/00006123-200206000-00033 12015860

[22] Sugiu K, Tokunaga K, Watanabe K, et al Emergent endovascular treatment of ruptured vertebral artery dissecting aneurysms. Neuroradiology 2005;47:158–64. 10.1007/s00234-005-1341-4 15703929

[23] Yasui T, Komiyama M, Nishikawa M, et al Subarachnoid hemorrhage from vertebral artery dissecting aneurysms involving the origin of the posteroinferior cerebellar artery: report of two cases and review of the literature. Neurosurgery 2000;46:196–200. 10.1093/neurosurgery/46.1.196 10626950

[24] Redekop G, TerBrugge K, Willinsky R Subarachnoid hemorrhage from vertebrobasilar dissecting aneurysm treated with staged bilateral vertebral artery occlusion: the importance of early follow-up angiography: technical case report. Neurosurgery 1999;45:1258–63. 10.1097/00006123-199911000-00056 10549948

[25] Wilkinson DA, Wilson TJ, Stetler WR, et al Subarachnoid haemorrhage with bilateral intracranial vertebral artery dissecting aneurysms treated by staged endovascular stenting. Case Reports 2013;2013:bcr0320126002 10.1136/bcr-03-2012-6002 PMC3603424 23417929

[26] Andic C, Aydemir F, Kardes O, et al Single-Stage endovascular treatment of multiple intracranial aneurysms with combined endovascular techniques: is it safe to treat all at once? J Neurointerv Surg 2017;9:1069–74. 10.1136/neurintsurg-2016-012745 27977003

[27] Kim M, Levy EI, Meng H, et al Quantification of hemodynamic changes induced by virtual placement of multiple stents across a wide-necked basilar trunk aneurysm. Neurosurgery 2007;61:1305–12. 10.1227/01.neu.0000306110.55174.30 18162911 PMC2756037

[28] Zenteno MA, Santos-Franco JA, Freitas-Modenesi JM, et al Use of the sole stenting technique for the management of aneurysms in the posterior circulation in a prospective series of 20 patients. J Neurosurg 2008;108:1104–18. 10.3171/JNS/2008/108/6/1104 18518712

[29] Guan J, Li G, Kong X, et al Endovascular treatment for ruptured and unruptured vertebral artery dissecting aneurysms: a meta-analysis. J Neurointerv Surg 2017;9:558–63. 10.1136/neurintsurg-2016-012309 27220870

[30] Chalouhi N, Tjoumakaris S, Dumont AS, et al Treatment of posterior circulation aneurysms with the pipeline embolization device. Neurosurgery 2013;72:833–89. 10.1227/NEU.0b013e31828ba984 23407289

[31] Albuquerque FC, Park MS, Abla AA, et al A reappraisal of the pipeline embolization device for the treatment of posterior circulation aneurysms. J Neurointerv Surg 2015;7:641–5. 10.1136/neurintsurg-2014-011340 25092926

[32] Tan LA, Keigher KM, Munich SA, et al Thromboembolic complications with pipeline embolization device placement: impact of procedure time, number of stents and pre-procedure P2Y12 reaction unit (Pru) value. J Neurointerv Surg 2015;7:217–21. 10.1136/neurintsurg-2014-011111 24553344

[33] Ishikawa T, Yamaguchi K, Anami H, et al Stent-assisted coil embolisation for bilateral vertebral artery dissecting aneurysms presenting with subarachnoid haemorrhage. Neuroradiol J 2016;29:473–8. 10.1177/1971400916666559 27558993 PMC5131767

[34] Komotar RJ, Mocco J, Samuelson RM, et al Rapidly successive, symptomatic, bilateral, spontaneous vertebral artery dissections: treatment with stent reconstruction. Surg Neurol 2009;72:300–5. 10.1016/j.surneu.2008.02.029 18514287

